# Retraction Note: ITGA3 acts as a purity-independent biomarker of both immunotherapy and chemotherapy resistance in pancreatic cancer: bioinformatics and experimental analysis

**DOI:** 10.1007/s10142-024-01373-4

**Published:** 2024-05-11

**Authors:** Xiaohao Zheng, Yongxing Du, Mingyang Liu, Chengfeng Wang

**Affiliations:** 1 Department of Pancreatic and Gastric Surgery, National Cancer Center/National Clinical Research Center for Cancer/Cancer Hospital, Chinese Academy of Medical Sciences and Peking Union Medical College, Beijing, 100021 China; 2 State Key Lab of Molecular Oncology, National Cancer Center/National Clinical Research Center for Cancer/Cancer Hospital, Chinese Academy of Medical Sciences and Peking Union Medical College, Beijing, 100021 China; 3 Department of General Surgery, Shanxi Province Cancer Hospital/Shanxi Hospital Affiliated to Cancer Hospital, Chinese Academy of Medical Sciences/Cancer Hospital Affiliated to Shanxi Medical University, Taiyuan, Shanxi 030013 China

**Retraction Note: Functional & Integrative Genomics (2023) 23:196**

10.1007/s10142-023-01122-z

The Editor-in-Chief and the publisher have retracted this article. The article was submitted to be part of a guest-edited issue. An investigation by the publisher found a number of articles, including this one, with a number of concerns, including but not limited to compromised editorial handling and peer review process, inappropriate or irrelevant references or not being in scope of the journal or guest-edited issue. Based on the investigation’s findings, the Editor-in-Chief therefore no longer has confidence in the results and conclusions of this article.

The author, Xiaohao Zheng, has stated that all authors disagree with this retraction

